# Supplementary figures and images for: Cancer-associated IDH mutations induce Glut1 expression and glucose metabolic disorders through a PI3K/Akt/mTORC1-Hif1α axis
Source: PLoS One. 2021 Sep 13;16(9):e0257090. doi: 10.1371/journal.pone.0257090 (PMC8437293; doi:10.1371/journal.pone.0257090)

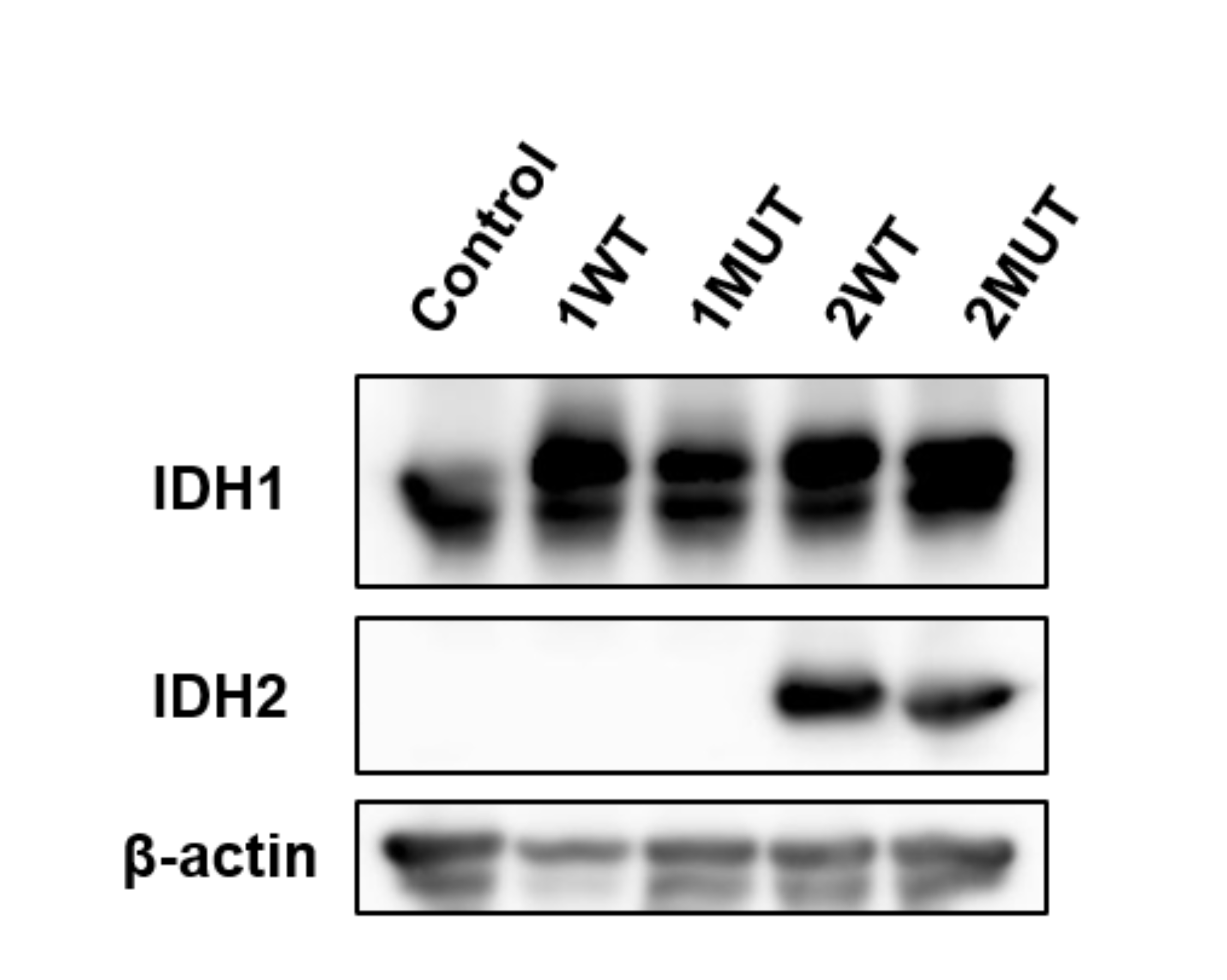

Supplement: S1 Fig — Western blot analysis of the MEF-1WT, MEF-1MUT, MEF-2WT, MEF-2MUT and control MEF cells. Expression of β-actin served as an internal control. The anti-IDH1 antibody recognized not only endogenously and exogenously expressed IDH1 but also exogenously expressed IDH2. The anti-IDH2 antibody recognized both endogenous and exogenous expression of IDH2. Expression of endogenous IDH2 is too low to be detected in the MEF cells. (TIF) [file pone.0257090.s001.tif]

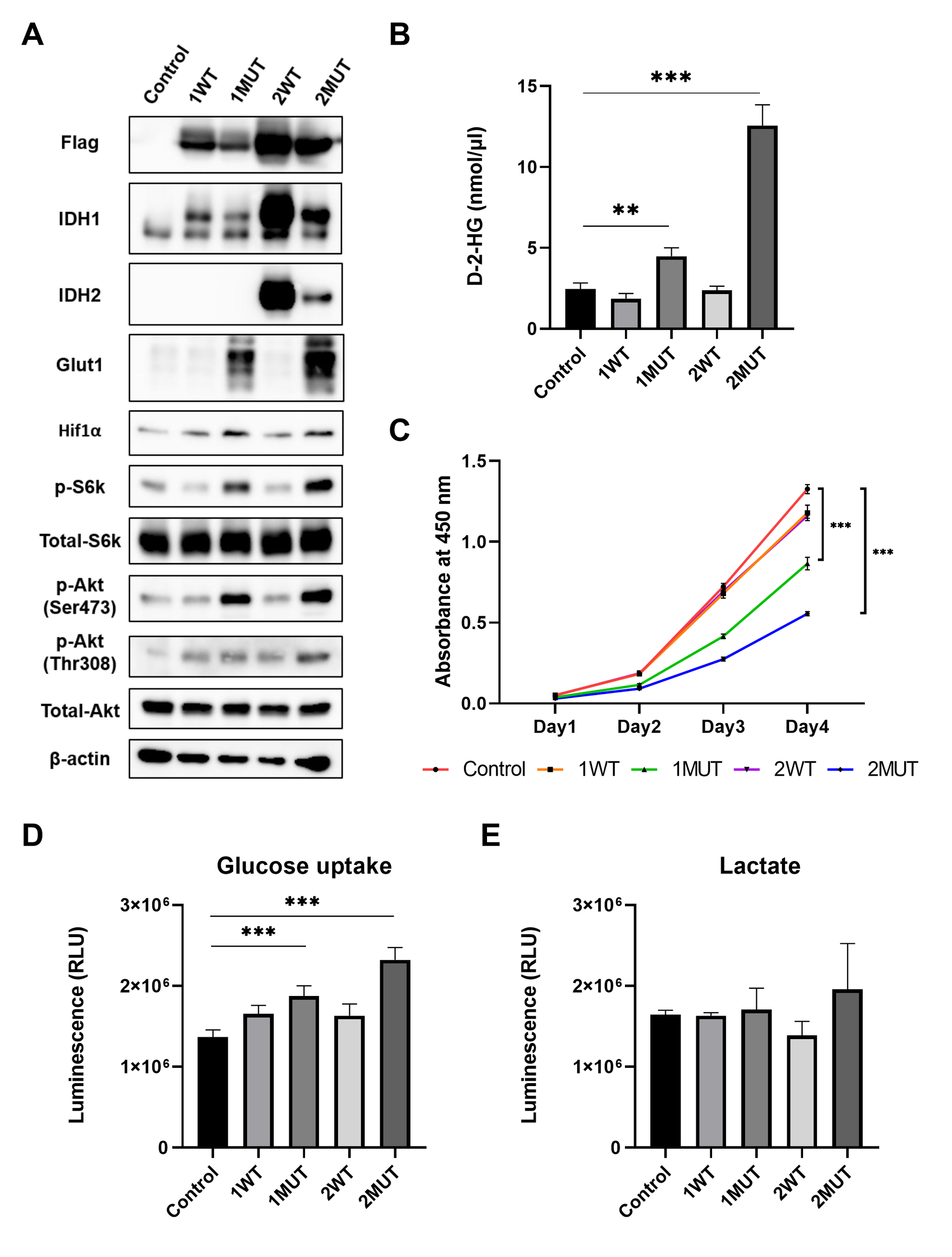

Supplement: S2 Fig — (A) Western blot analysis of the HCT116-1WT, HCT116-1MUT, HCT116-2WT, HCT116-2MUT and control HCT116 cells. Expression of β-actin served as an internal control. (B) Concentration of 2-HG in the lysates from cells indicated in (A). The data represent mean ± SD of three independent experiments. ** p<0.01, *** p<0.001. (C) The proliferation of cells was measured by WST-8 assay. The data represent mean ± SD of three independent experiments. *** p<0.001. (D) Glucose uptake levels in HCT116-1WT, HCT116-1MUT, HCT116-2WT, HCT116-2MUT, and the control cells were measured by a bioluminescent assay based on the detection of 2DG6P. The data represent mean ± SD of three independent experiments. *** p<0.001. (E) Intracellular lactate levels in the HCT116 cells indicated in (D) were measured by a bioluminescent assay for the detection of L-lactate. The data represent mean ± SD of three independent experiments. (TIF) [file pone.0257090.s002.tif]

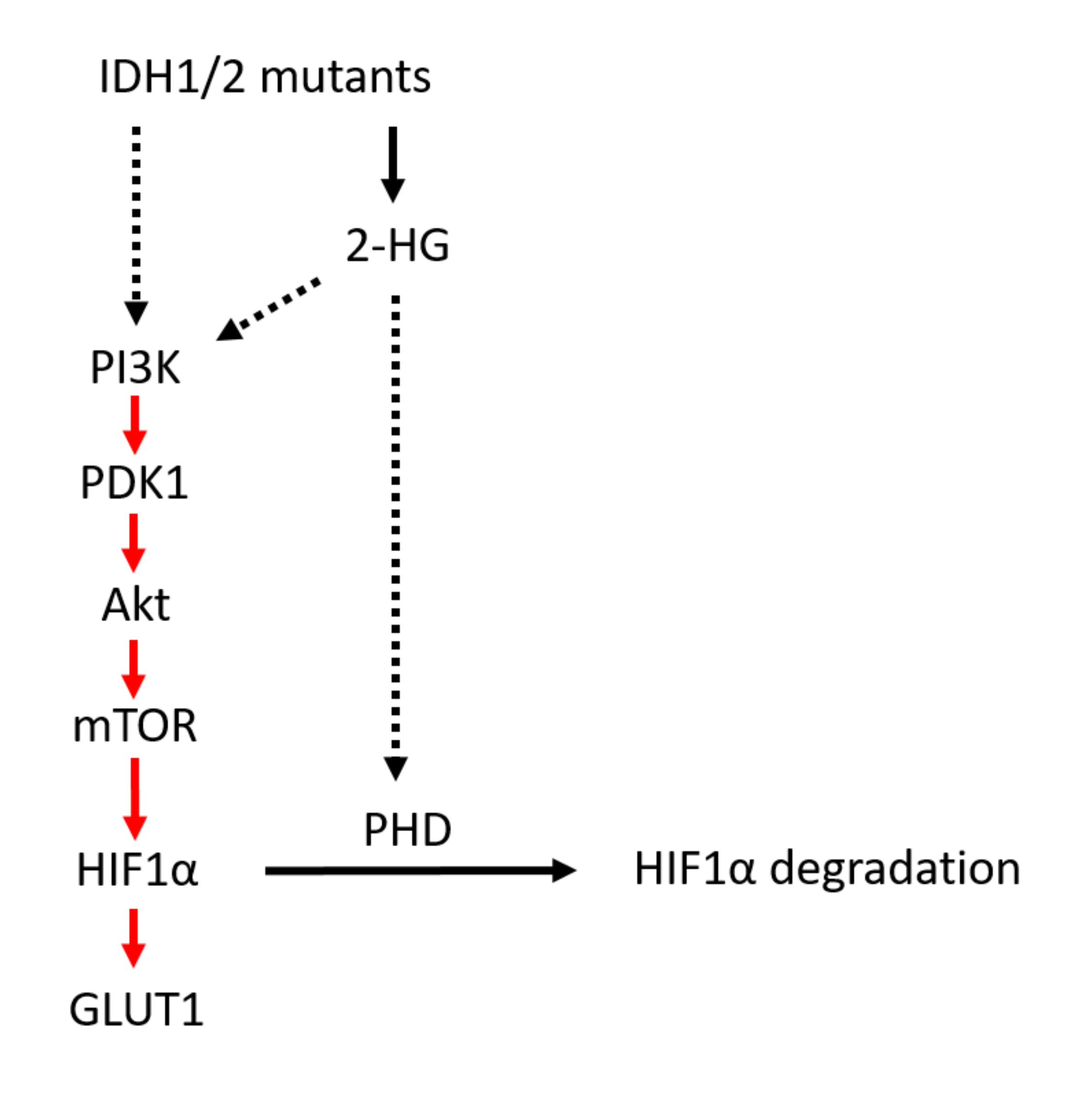

Supplement: S3 Fig — Red solid arrows indicate the mechanism elucidated in this research. Black solid arrows show regulatory effects reported previously. Black dotted arrows exhibit potential regulations remaining unknown. (TIF) [file pone.0257090.s003.tif]
