# Supplementary material for: Cancer-associated IDH mutations induce Glut1 expression and glucose metabolic disorders through a PI3K/Akt/mTORC1-Hif1α axis
Source: PLoS One. 2021 Sep 13;16(9):e0257090. doi: 10.1371/journal.pone.0257090 (PMC8437293; doi:10.1371/journal.pone.0257090)
Supplement: S1 Raw images — (PDF) [file pone.0257090.s007.pdf]

Fig 1A

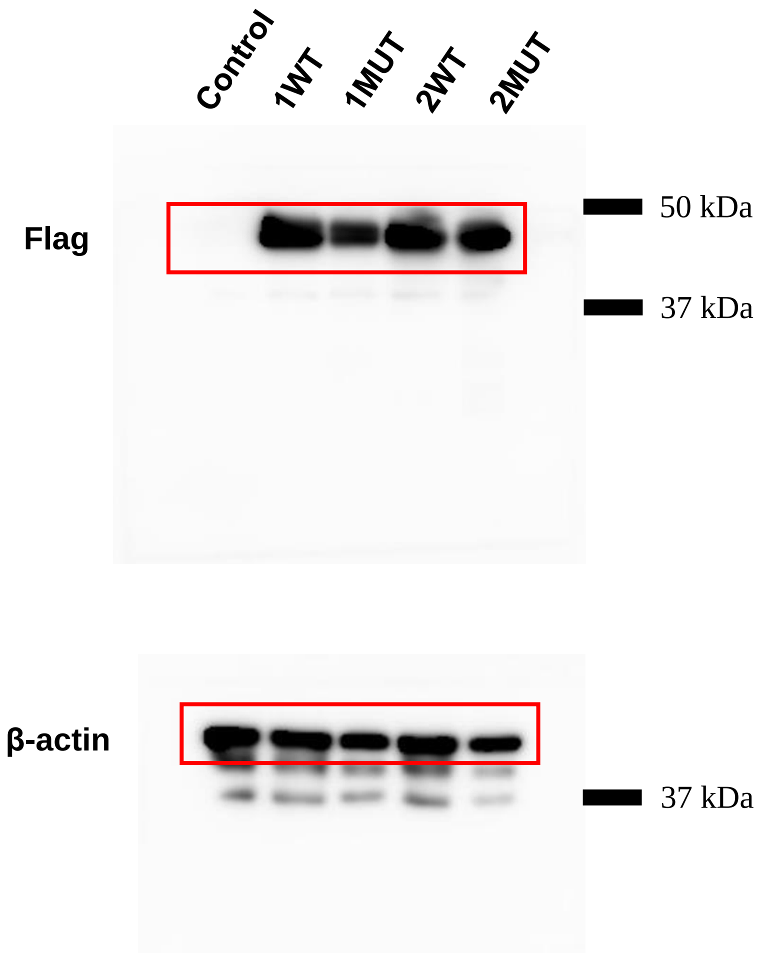
Control
1WT
1MUT
2WT
2MUT
Flag
50 kDa
37 kDa
β-actin
37 kDa

Fig 3G

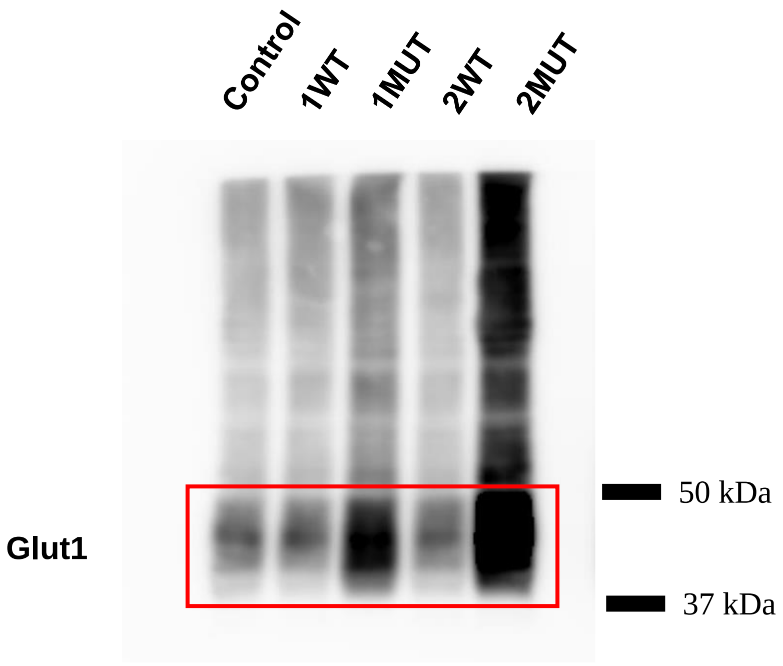
Control
1WT
1MUT
2WT
2MUT
Glut1
50 kDa
37 kDa

Fig 3G

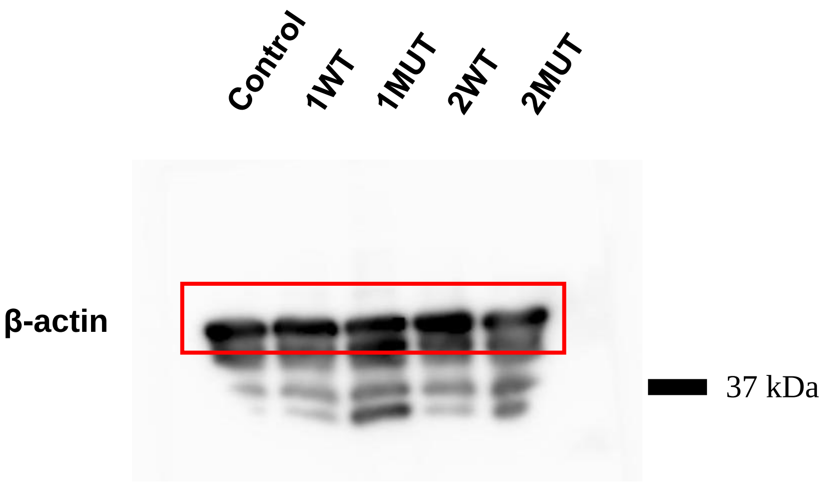

Control
1WT
1MUT
2WT
2MUT
β-actin
37 kDa

Fig 3H

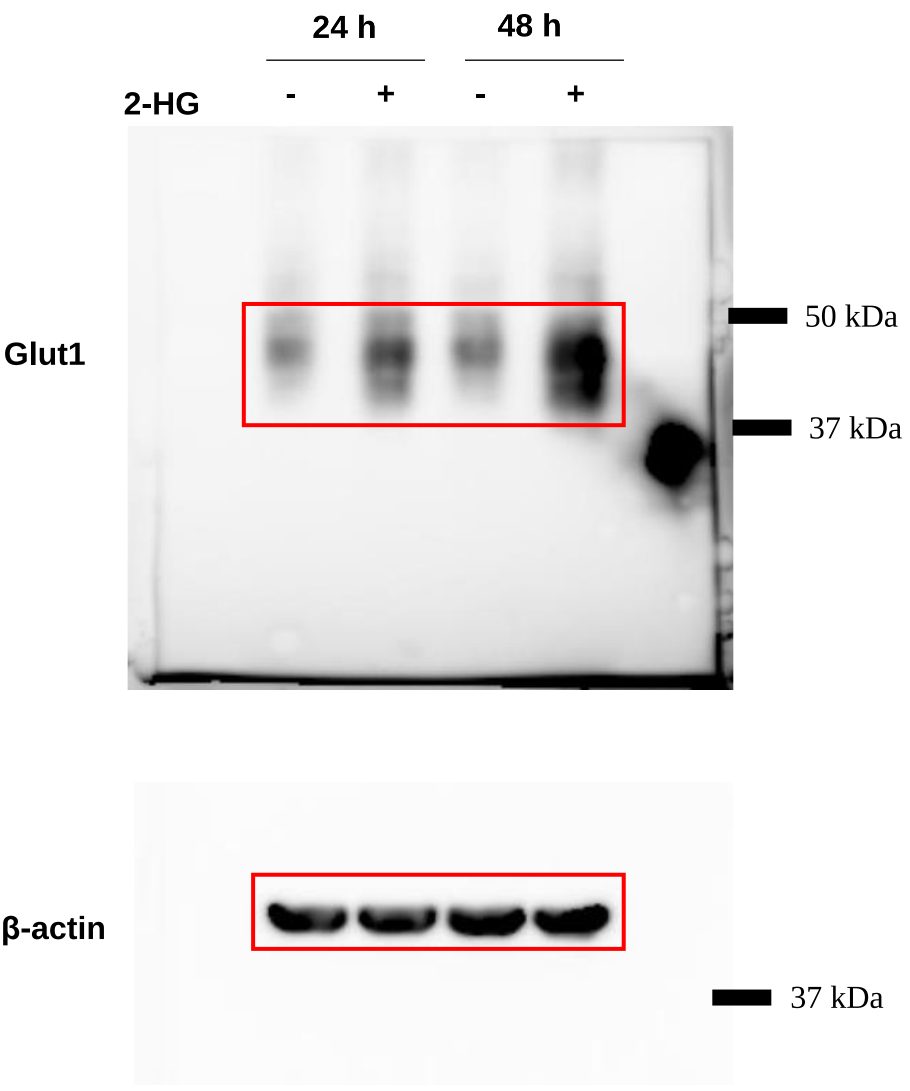

24 h
48 h
2-HG
-
+
-
+
Glut1
50 kDa
37 kDa
β-actin
37 kDa

Fig 5A (Using same lysate with Fig 1A)

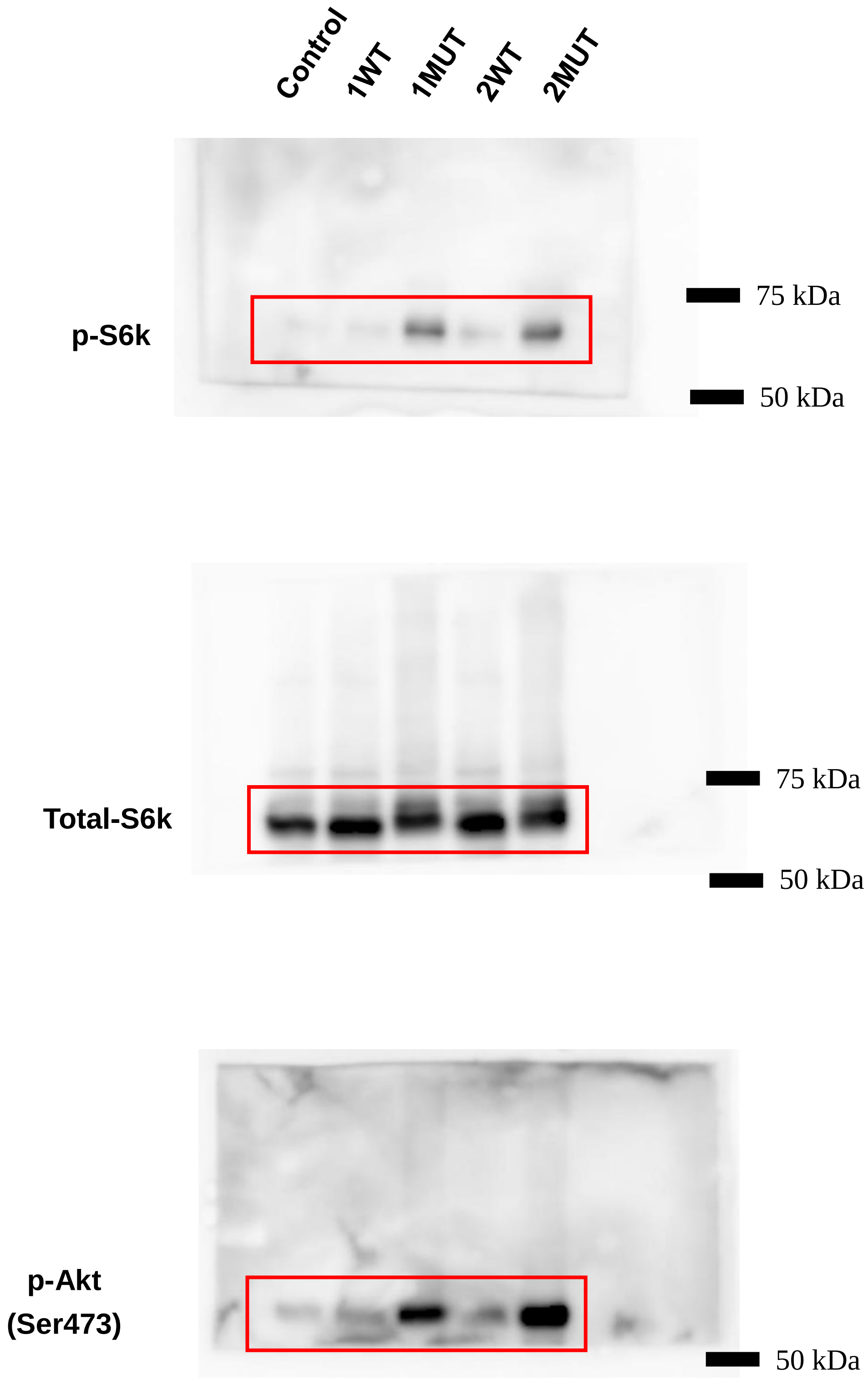

Fig 5A (Using same lysate with Fig 1A)

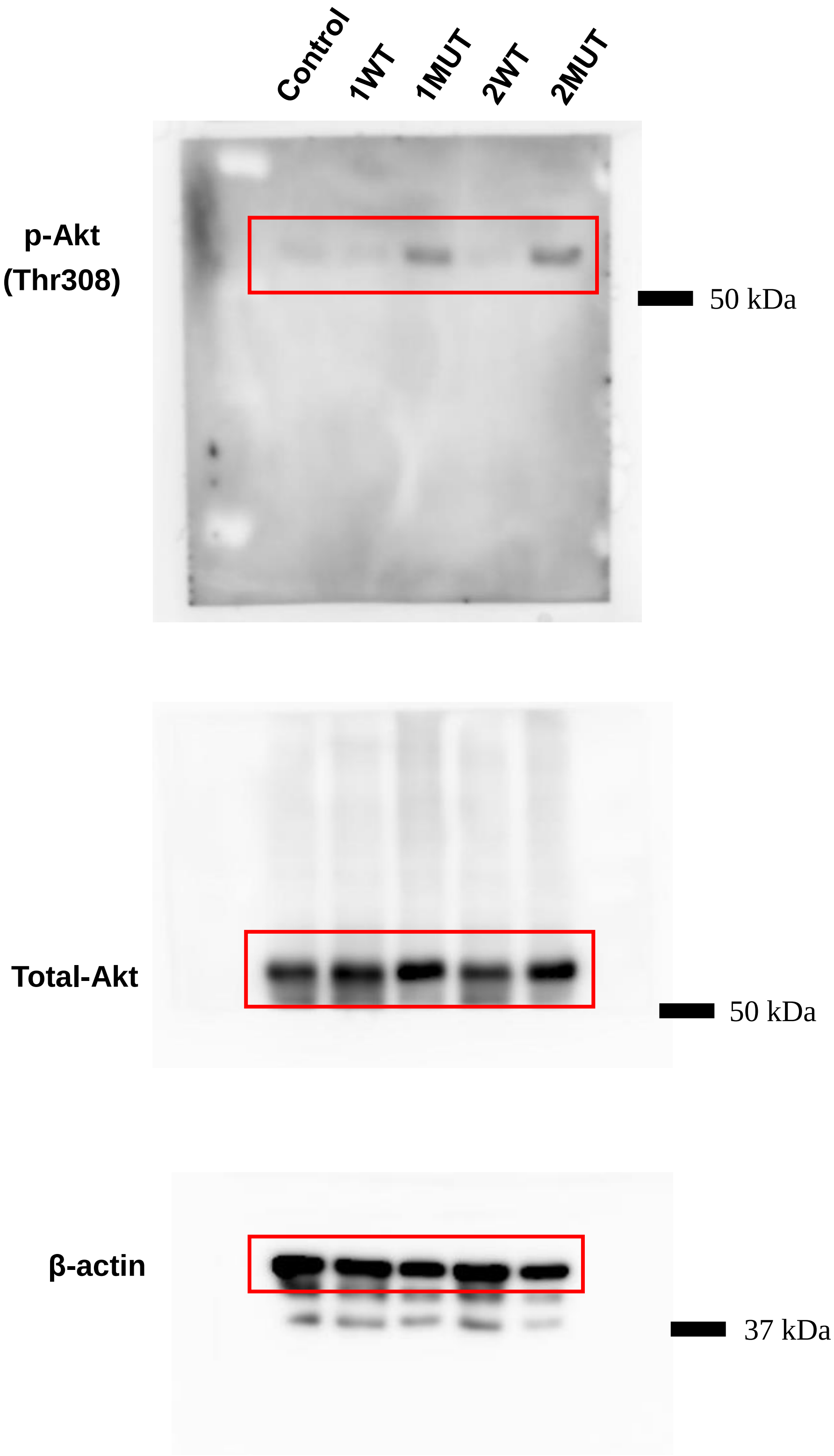

Fig 5B

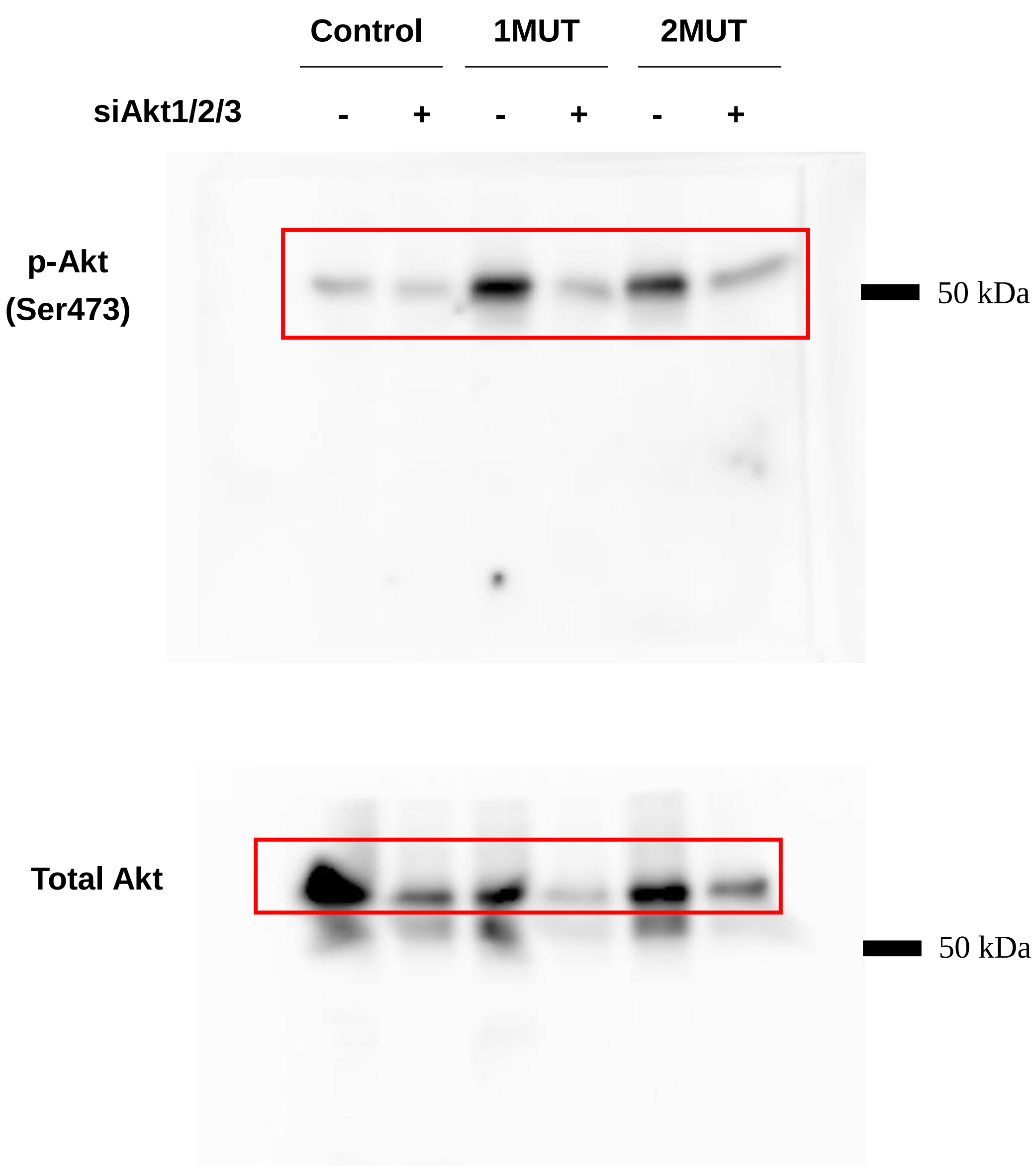
Control
1MUT
2MUT
siAkt1/2/3
- + - + - +
p-Akt
(Ser473)
50 kDa
Total Akt
50 kDa

Fig 5B

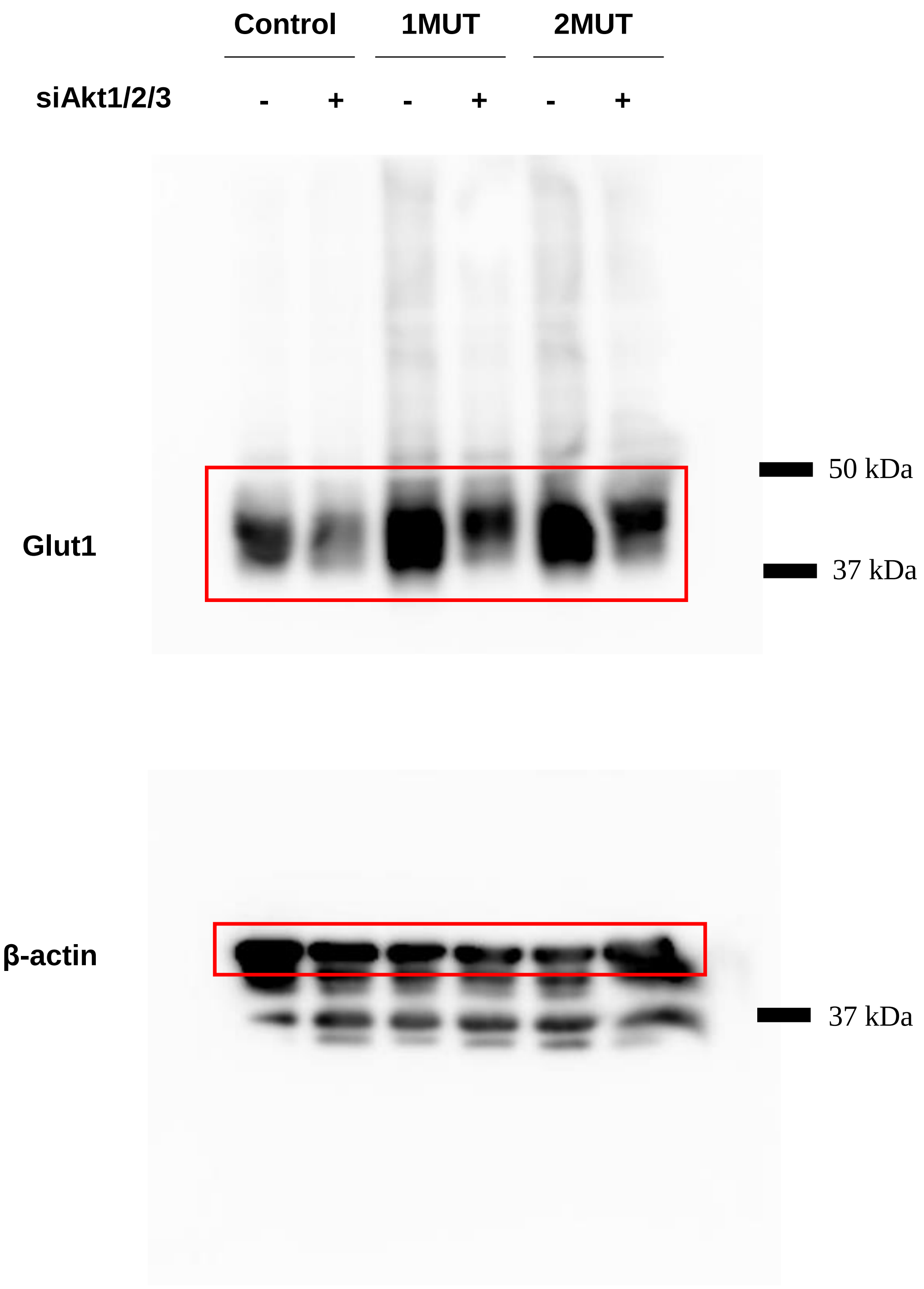

Fig 5C

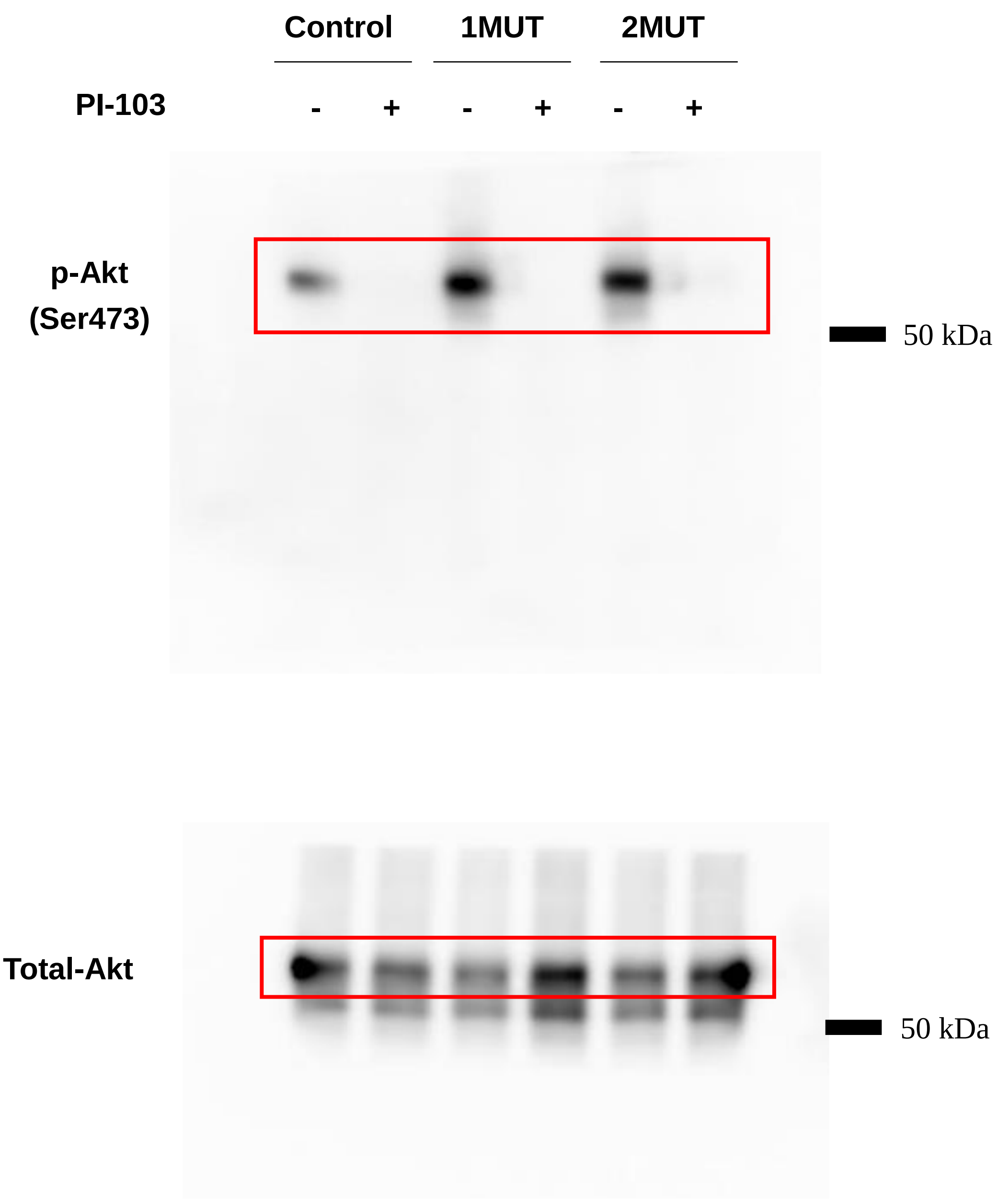

Fig 5C

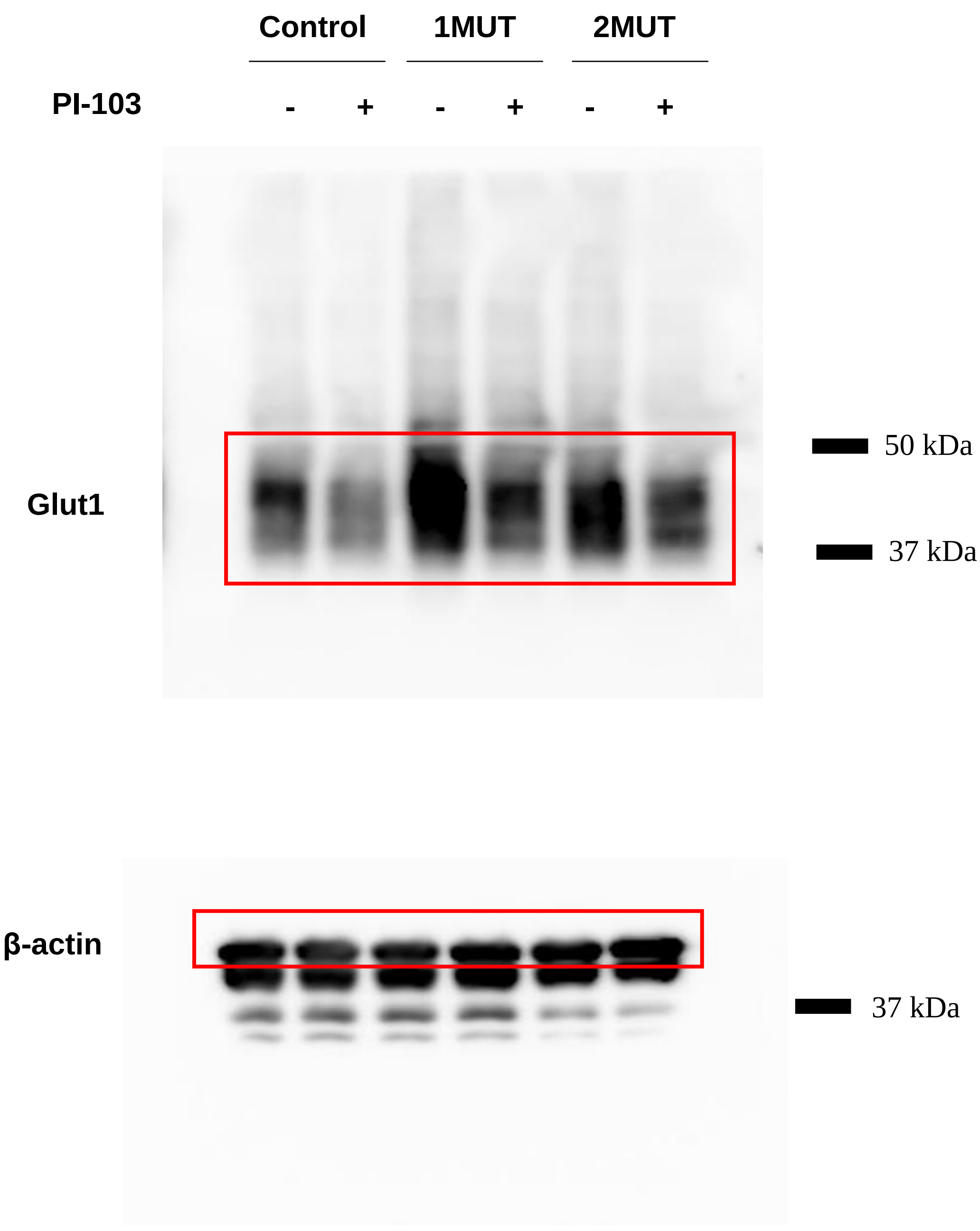

Fig 5D

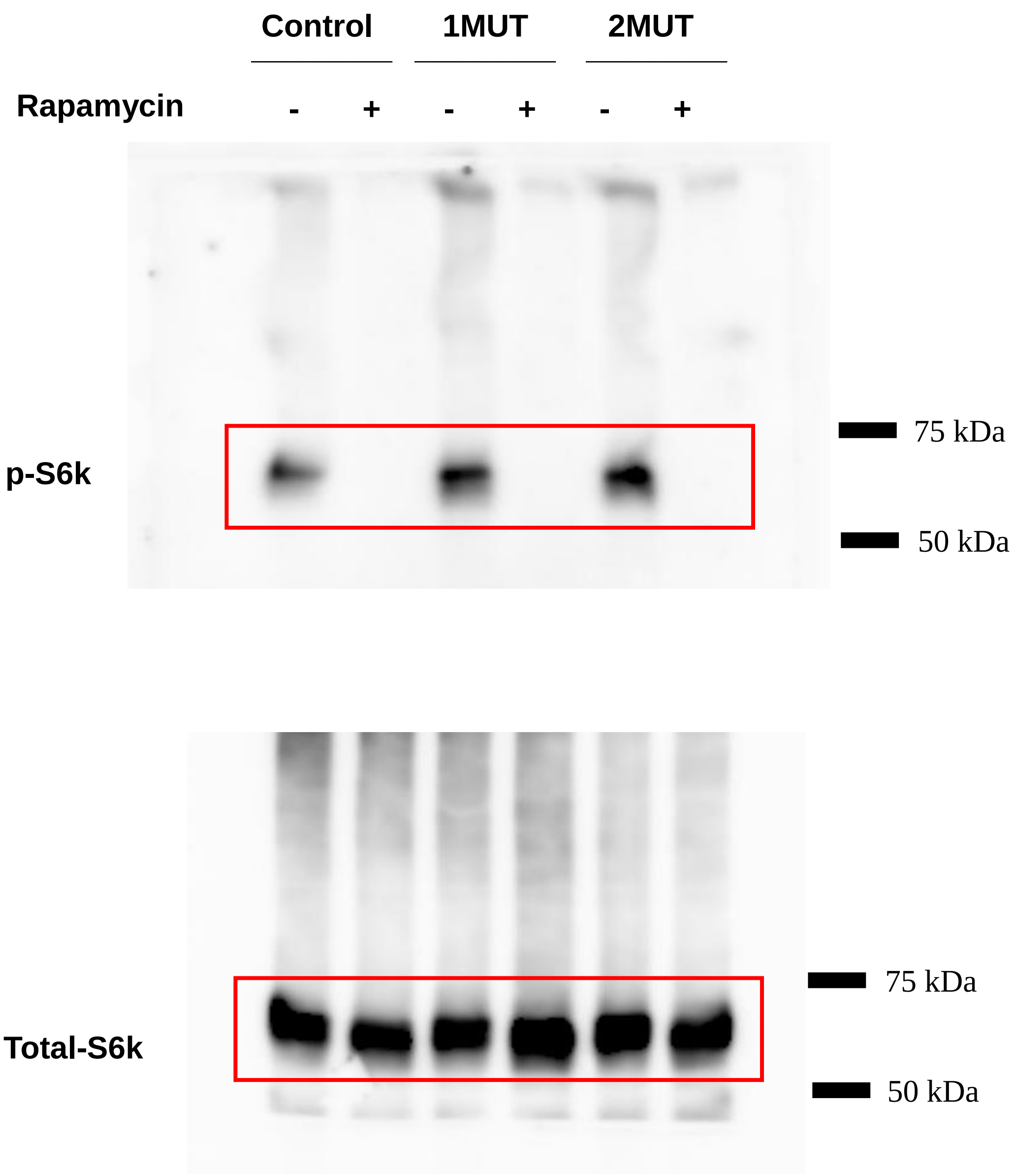

Control
1MUT
2MUT
Rapamycin
- + - + - +
p-S6k
75 kDa
50 kDa
Total-S6k
75 kDa
50 kDa

Fig 5D

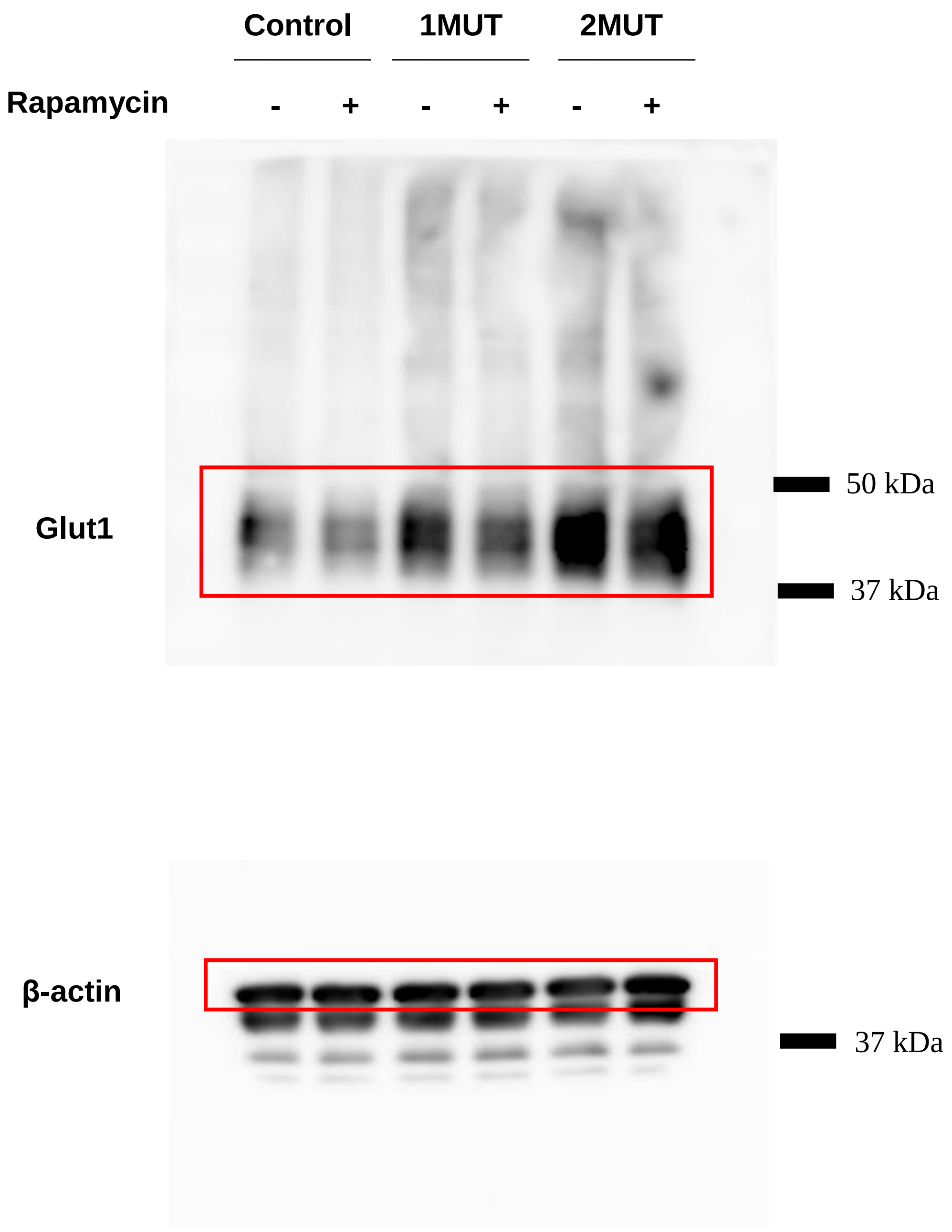
Control
1MUT
2MUT
Rapamycin
- + - + - +
Glut1
50 kDa
37 kDa
β-actin
37 kDa

Fig 6A

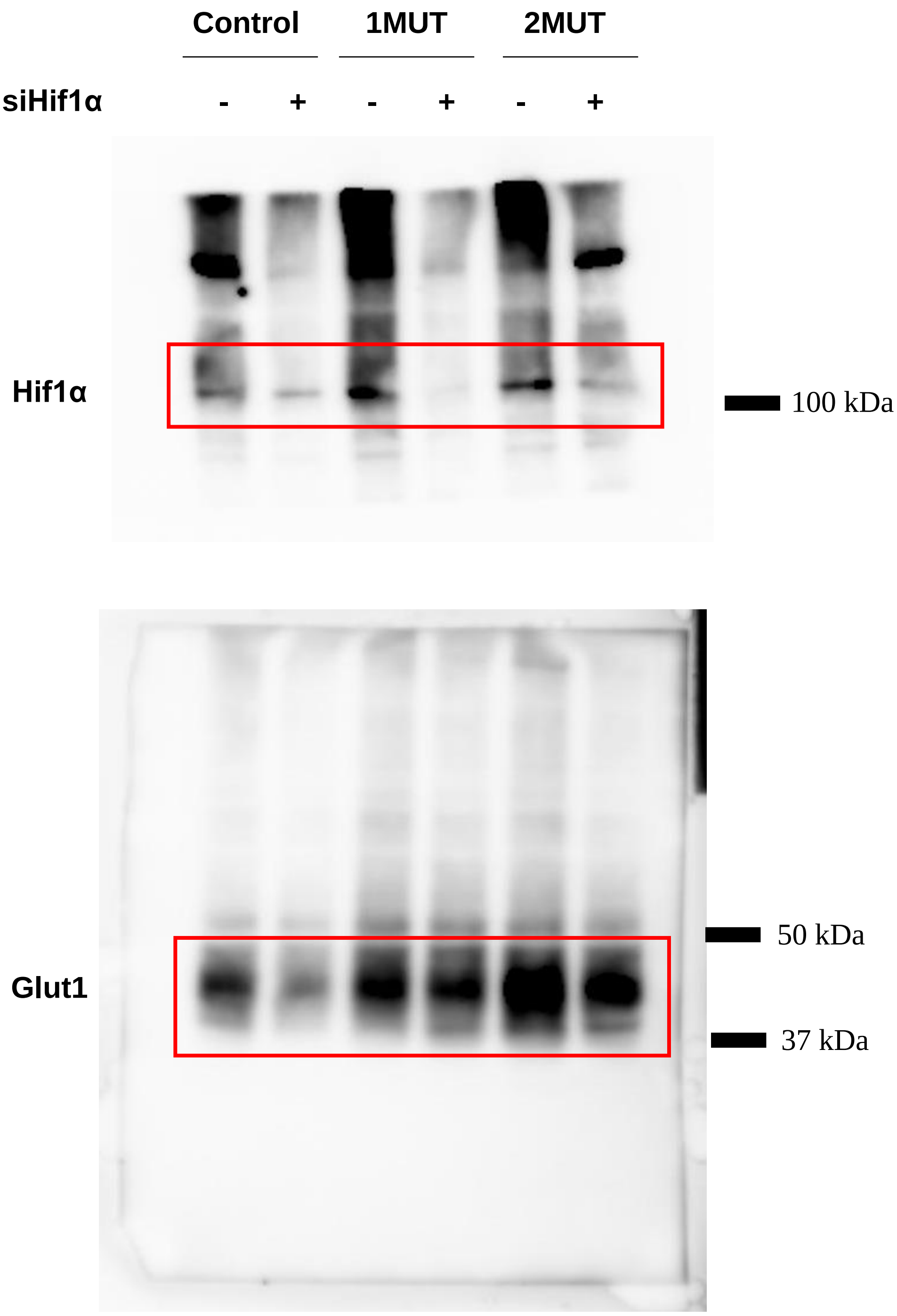

Fig 6A

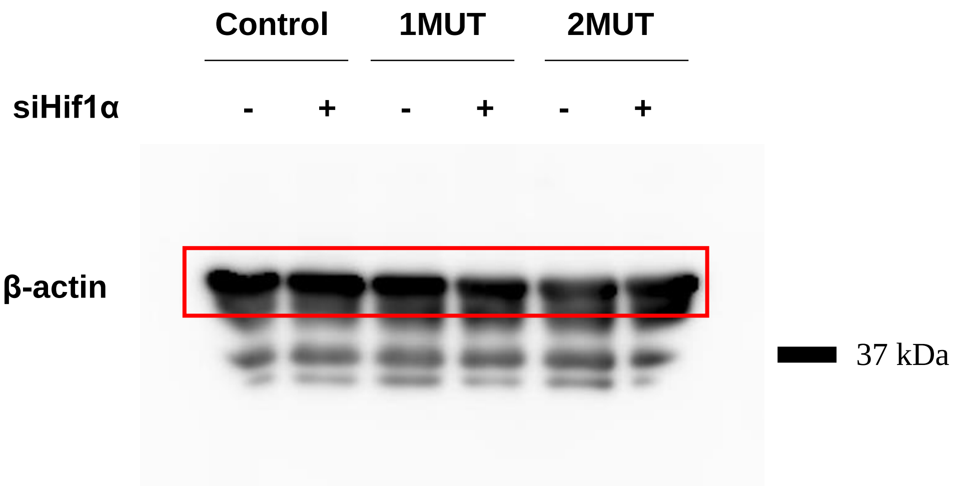
Control
1MUT
2MUT
siHif1α
- + - + - +
β-actin
37 kDa

Fig 6C

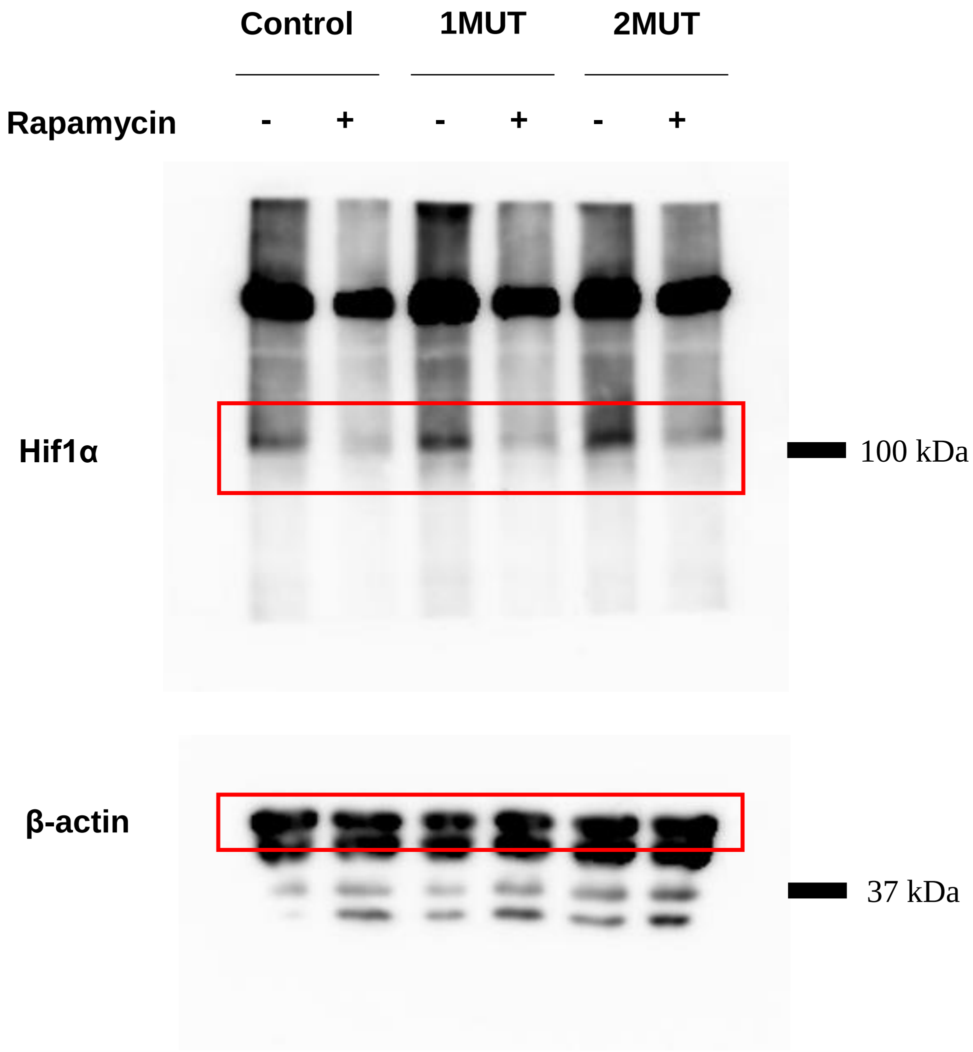
Control
1MUT
2MUT
Rapamycin
- + - + - +
Hif1α
100 kDa
β-actin
37 kDa

S1 Fig

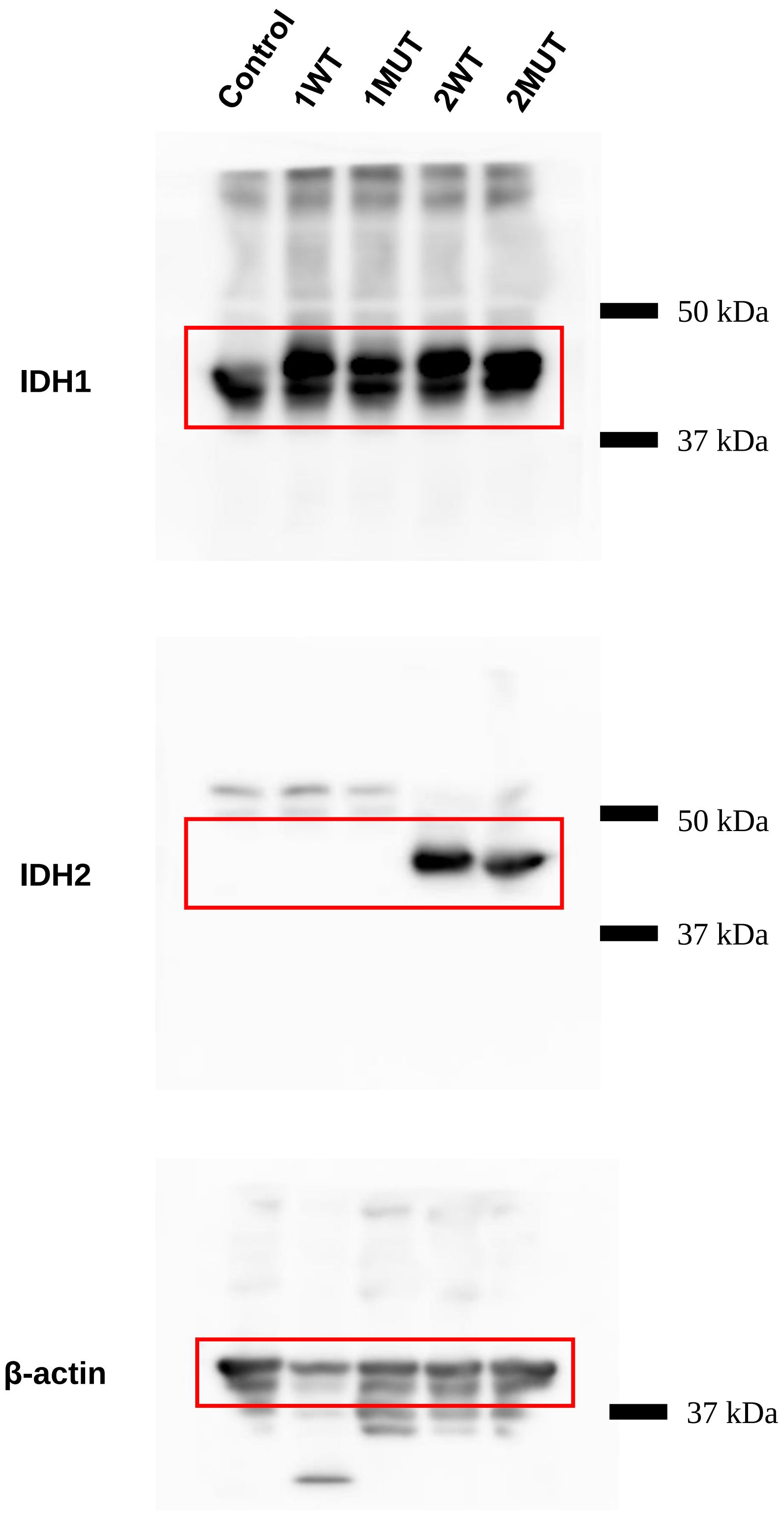
Control
1WT
1MUT
2WT
2MUT
IDH1
50 kDa
37 kDa
IDH2
50 kDa
37 kDa
β-actin
37 kDa

S2 Fig A

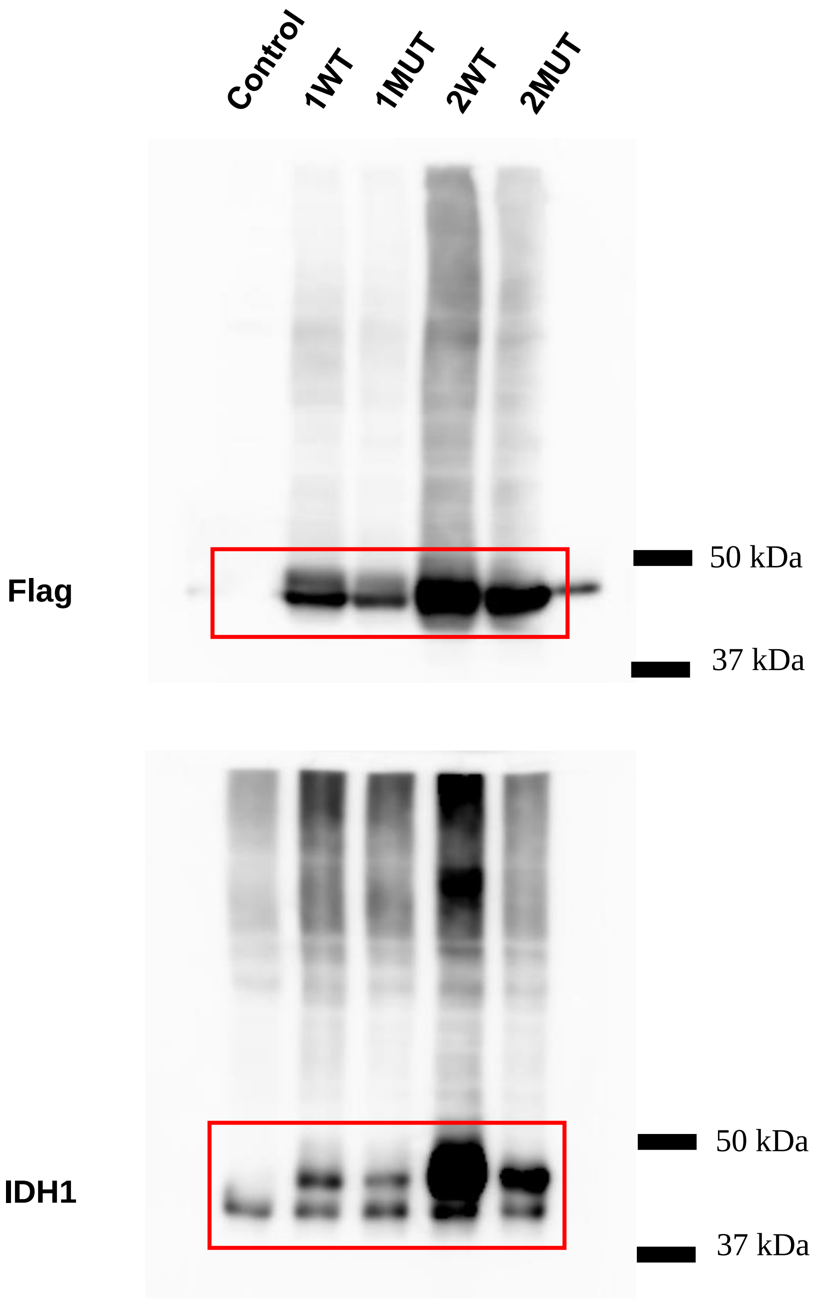

S2 Fig A

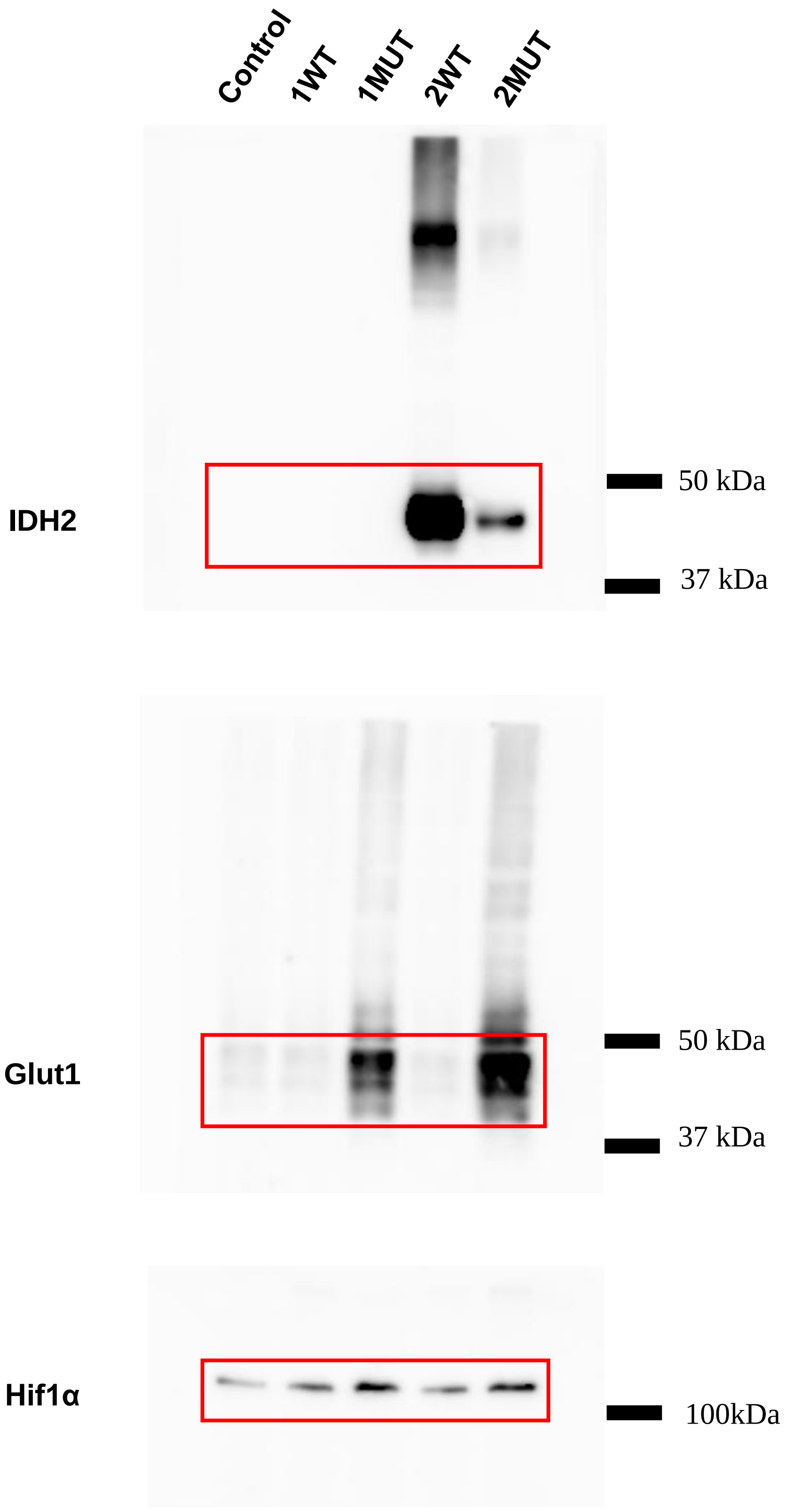
Control
1WT
1MUT
2WT
2MUT
IDH2
50 kDa
37 kDa
Glut1
50 kDa
37 kDa
Hif1α
100kDa

S2 Fig A

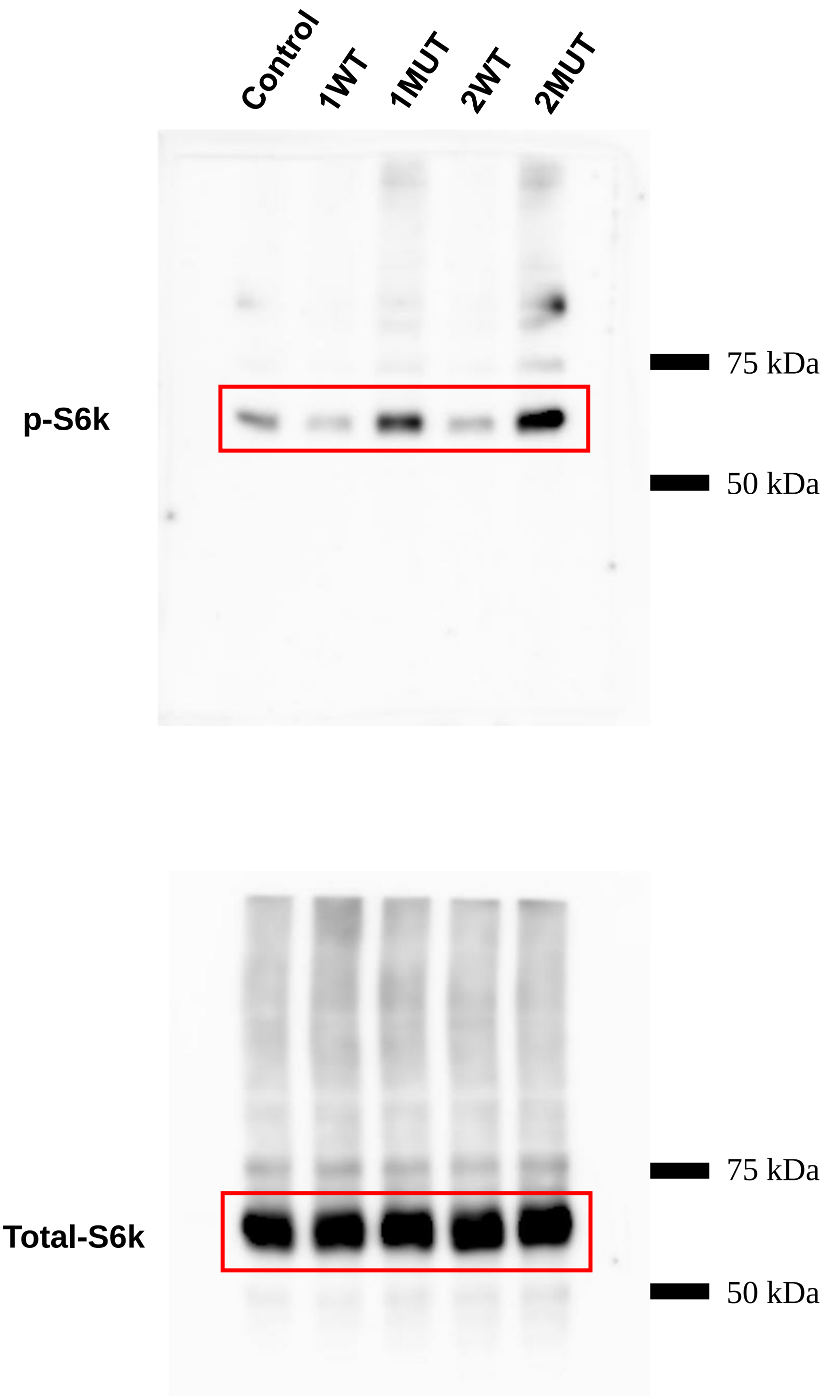
Control
1WT
1MUT
2WT
2MUT
p-S6k
75 kDa
50 kDa
Total-S6k
75 kDa
50 kDa

S2 Fig A

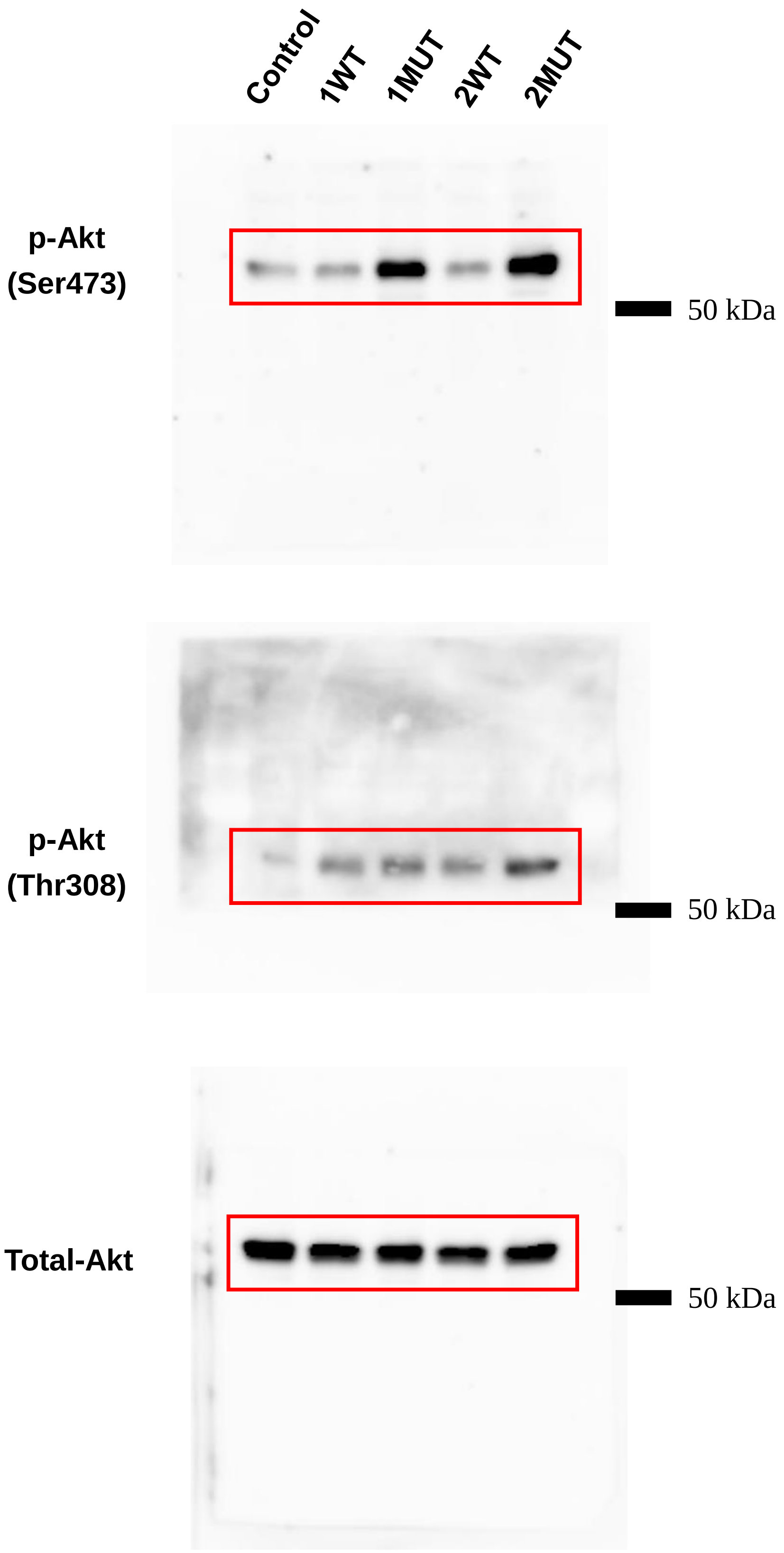
Control
1WT
1MUT
2WT
2MUT
p-Akt
(Ser473)
50 kDa
p-Akt
(Thr308)
50 kDa
Total-Akt
50 kDa

S2 Fig A

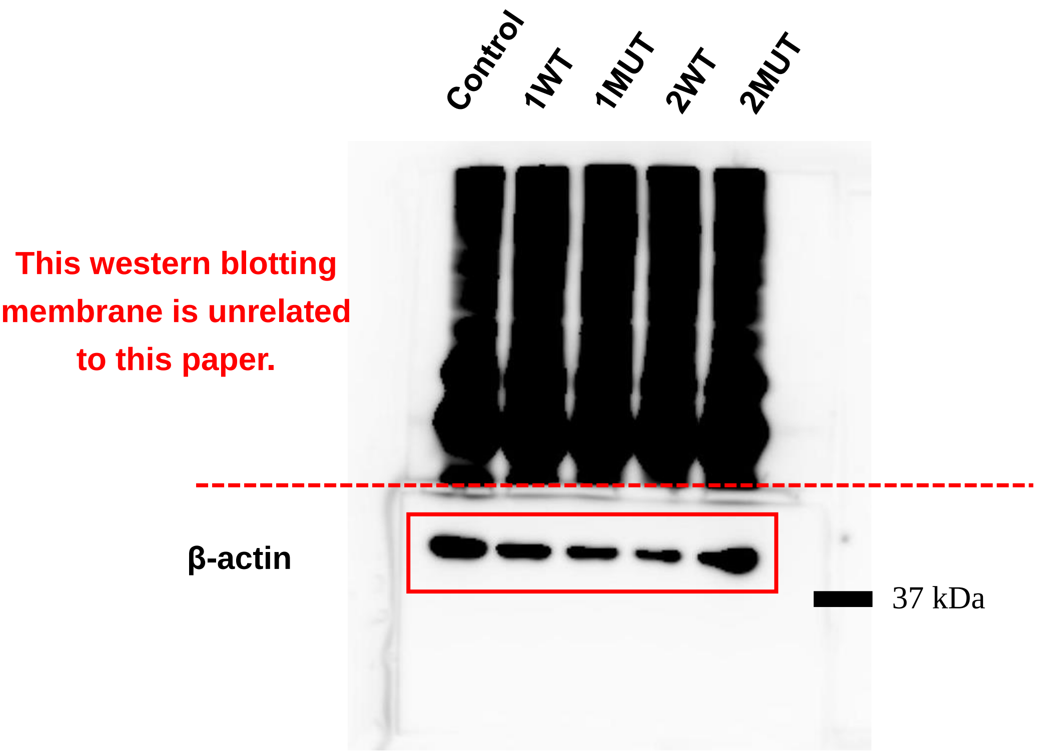
Control
1WT
1MUT
2WT
2MUT
This western blotting membrane is unrelated to this paper.
β-actin
37 kDa
