# Supplementary material for: Cancer-associated IDH mutations induce Glut1 expression and glucose metabolic disorders through a PI3K/Akt/mTORC1-Hif1α axis
Source: PLoS One. 2021 Sep 13;16(9):e0257090. doi: 10.1371/journal.pone.0257090 (PMC8437293; doi:10.1371/journal.pone.0257090)
Supplement: Attachment — Submitted filename: Response to Reviewers.docx [file pone.0257090.s008.docx]

**Reply to the comments**

We sincerely thank the editor and reviewers for their thoughtful and positive comments to our manuscript.

To editor:

As the editor suggested, to generalize the findings that we obtained using MEF cells, we generated HCT116 cells, a human colorectal cancer cell line, stably expressing mutant IDH1 and IDH2, and evaluated the 2-HG accumulation, cell proliferation, genes expression and metabolic characteristics in these cells as additional experiments.

We found that IDH1/2 mutations suppressed cell proliferation HCT116 cells as well as MEF cells. As we noted in the discussion section, although IDH mutations had been reported to promote cell proliferation in many types of cancer cells as a basal oncogenic activity, other studies also showed contradictory results of reduced cell growth induced by IDH mutations or 2-HG. Here, we demonstrated that IDH mutations suppress cell proliferation of HCT116 cells.

IDH1/2 mutations induced accumulation of 2-HG, activation of PI3K/Akt/mTORC1-Hif1α axis, and enhancement of glucose uptake in HCT116 cells, all of which were consistent to the findings obtained in MEF cells, except the lactate production. We speculated that the enhanced Warburg effect in HCT116 cells reported in previous studies might weaken the effect of IDH mutants on glycolysis and made it difficult to detect the change of lactate levels induced by IDH1/2 mutations.

According to the editor’s suggestion, we have deposited the raw data to GEO database (GSE180369).

Furthermore, according to the editor’s constructive suggestion, we added a schematic of the underlying mechanism of Glut1 expression regulated by mutant IDH1/2 through the PI3K/Akt/mTORC1-Hif1α axis in supplementary Figure 3.

To reviewer #1:

1. We thank the reviewer’s comment. As the reviewer mentioned, we did not show the effect of exogenous expression of cancer-associated *IDH1* and *IDH2* mutations in cancer cells. Therefore, we generated HCT116 cells, a human colorectal cancer cell line, stably expressing mutant IDH1 and IDH2, and evaluated the 2-HG accumulation, cell proliferation, genes expression and metabolic characteristics in these cells as additional experiments.
2. We thank the reviewer’s constructive comment. Actually, we preserved the total RNA samples of MEF cells stably expressing mutant IDH1 (MEF-1MUT) when we performed RNA-Seq on the *IDH2* mutation group (MEF-2MUT). Since our RNA-seq before aimed to clarify the effect of IDH mutant through accumulated 2-HG, we finally determined to use RNA sample of MEF-2MUT, whose 2-HG accumulation is significantly higher than MEF-1MUT. As a result, *Slc2a1*, encoding Glut1, was up-regulated in MEF-2MUT and 2-HG treated MEF cells. This time, we carried out RNA-seq in MEF-1MUT using the samples we kept before. Unfortunately, although we found increased read counts of *Slc2a1* in MEF-1MUT cells compare to the control cells (Data showed below), there was no statistical significance between two groups. However, as shown in Figure 3C and 3G, both real-time PCR and western blotting results demonstrated that the *Slc2a1* expression was up-regulated in both MEF-1MUT and MEF-2MUT. Therefore, a common regulatory mechanism for *Slc2a1* should be induced by IDH1 and IDH2 mutants.
3. According to the reviewer’s comment, we analyzed glucose uptake and lactate production in HCT116 cells. As we expected, both mutant IDH1 and IDH2 significantly increased glucose uptake in HCT116 cells. However, lactate production seemed to be unaltered. Previous research reported that lactate production was augmented in HCT116 cells. This may suggest that the enhanced Warburg effect in HCT116 cells weakened the effect on glycolysis by IDH mutants and made it difficult to detect the change of lactate levels induced by IDH1/2 mutations. We mentioned this issue in the discussion section.
4. According to the reviewer’s comment, we added the western blotting results of IDH1 and IDH2 in supplementary Figure 1.
5. According to the reviewer’s comment, we changed the results in Figure 1B into new data of 2-HG measurement with statistical analysis. The data represent mean ± SD of three independent experiments.
6. According to the reviewer’s comment, we added the statement on the cutoff for DEG determination utilized in our RNA-seq analysis in the Materials and Methods section.
7. We thank the reviewer’s thoughtful suggestion. Real-time PCR results in Figure 6B showed that Hif1α mRNA was suppressed by rapamycin, whereas in Figure 6C, rapamycin markedly decreased the Hif1α protein expression. This difference between RNA and protein expression led us to the inference that post-transcriptional regulation may play a major role in the expression of Hif1α in context of *IDH1/2* mutations. However, as the reviewer pointed out, we did not have any more evidence to support our speculation. Therefore, we agree that the sentence seems to be an overstatement and consider that both transcriptional and post-transcriptional may play a role in the expression of Hif1α in the presence of *IDH1/2* mutations. We changed the description in the discussion section.
8. In Figure 6A, we showed that Glut1 expression levels were reduced after Hif1α knockdown in MEF-1MUT and MEF-2MUT cells. We think these results suggest that IDH1/2 regulates Glut1 expression through Hif1α.
9. According to the reviewer’s suggestion, we have already deposited our RNA-seq data to GEO database (GSE180369).

To reviewer #2:

Comment on ‘Methods’

We thank the reviewer’s thoughtful comments on the methods of this study. Data in this paper actually is the *in vitro* part of a study aimed to establish and analysis of a novel mouse model of human cancer based on knockin of cancer-associated *IDH1/2* mutations (Data not published). For the study of the functions of *IDH1/2* mutations in tumorigenesis, it is desirable to use normal epithelial cells instead of cancer cells to exclude the influence of complex genetic and metabolic background in cancer cells. However, we did not have any available normal epithelial cells. Therefore, we chose MEF cells in this research. Organoids established from normal epithelial tissues can be utilized in future studies. The control MEF cells means MEF cells stably expressing pMX-control vector, officially named pMXs-puro vector.

Comment on ‘Results’

We thank the reviewer’s thoughtful comment of the results of this study. As the reviewer mentioned, VEGF pathway is a downstream pathway mainly regulated by HIF-1 pathway. Although genes involved in these two pathways are not necessarily common, Hif1α is one of the genes that are involved in both pathways. The GSEA based on our RNA-seq showed that genes associated with “HIF-1 pathway” were enriched in MEF cells expressing mutant IDH2 compared to the control cells, and the genes correlated with “VEGF pathway” were enriched by 2-HG treatment in MEF cells. These results indicated that Hif1α might be regulated by *IDH* mutation through 2-HG. Further, we proved our hypothesis that both transcriptional and post-transcriptional regulation play a role in the expression of Hif1α in the presence of IDH1/2 mutations using qRT-PCR and western blotting.
